# Circulating Biomarkers of Immune Activation, Oxidative Stress and Inflammation Characterize Severe Canine Visceral Leishmaniasis

**DOI:** 10.1038/srep32619

**Published:** 2016-09-06

**Authors:** Manuela S. Solcà, Bruno B. Andrade, Melissa Moura Costa Abbehusen, Clarissa R. Teixeira, Ricardo Khouri, Jesus G. Valenzuela, Shaden Kamhawi, Patrícia Torres Bozza, Deborah Bittencourt Mothé Fraga, Valeria Matos Borges, Patrícia Sampaio Tavares Veras, Claudia Ida Brodskyn

**Affiliations:** 1 Laboratório de Patologia e Biointervenção, Instituto de Pesquisas Gonçalo Moniz, FIOCRUZ, 40296-710 Salvador, Brazil; 2 Laboratório Integrado de Microbiologia e Imunoregulação, Instituto de Pesquisas Gonçalo Moniz, FIOCRUZ, 40296-710 Salvador, Brazil; 3 Multinational Organization Network Sponsoring Translational and Epidemiological Research (MONSTER) Initiative, Fundação José Silveira, 40070-080 Salvador, Brazil; 4 Fundação Oswaldo Cruz, Fiocruz Piauí, 64128 Teresina, Brazil; 5 Vector Molecular Biology Section, Laboratory of Malaria and Vector Research, National Institute of Allergy and Infectious Diseases, National Institutes of Health, 12735 Twinbrook Parkway, Rockville, MD, 20852, USA; 6 Laboratório de Imunofarmacologia, Instituto Oswaldo Cruz, Bio-Manguinhos, FIOCRUZ, 21040-900 Rio de Janeiro, Brazil; 7 Departamento de Medicina Veterinária Preventiva e Produção Animal, Escola de Medicina Veterinária e Zootecnia, Universidade Federal da Bahia, 40170-110 Salvador, Brazil; 8 Instituto Nacional de Ciência e Tecnologia para Doenças Tropicais (INCT-DT), 40110-160 Salvador, Brazil; 9 Instituto Nacional de Ciência e Tecnologia de Investigação em Imunologia (III-INCT), 05403-900 São Paulo, Brazil

## Abstract

Clinical manifestations in canine visceral leishmaniasis (CVL) have not been clearly associated with immunological status or disease progression. We simultaneously assessed biomarkers of inflammation, immune activation, oxidative stress, and anti-sand fly saliva IgG concentrations in dog sera with different clinical manifestations to characterize a biosignature associated with CVL severity. In a cross-sectional exploratory study, a random population of 70 dogs from an endemic area in Brazil was classified according to CVL clinical severity and parasitological evaluation. A panel of biomarkers and anti–sand fly saliva IgG were measured in canine sera. Assessment of protein expression of profile biomarkers identified a distinct biosignature that could cluster separately animal groups with different clinical scores. Increasing severity scores were associated with a gradual decrease of LTB4 and PGE2, and a gradual increase in CXCL1 and CCL2. Discriminant analyses revealed that combined assessment of LTB4, PGE2 and CXCL1 was able to distinguish dogs with different clinical scores. Dogs with the highest clinical score values also exhibited high parasite loads and higher concentrations of anti-saliva antibodies. Our findings suggest CVL clinical severity is tightly associated with a distinct inflammatory profile hallmarked by a differential expression of circulating eicosanoids and chemokines.

Visceral leishmaniasis (VL) is a widespread disease caused by the protozoan *Leishmania infantum*. This parasite is transmitted to humans and animals through the bite of the infected sand fly *Lutzomyia longipalpis*^1^,^2^. Dogs are considered the main urban reservoir of the parasite and its presence in the endemic area is known as a risk factor for the occurrence of human VL^3^,^4^.

Clinical signs of canine VL (CVL) are non-specific, there is a widespread range of clinical manifestations varying from visceral to cutaneous presentation of the disease^5^,^6^, on the other hand some animals do not display any clinical signs during the course of infection^7^. The resistance and susceptibility to CVL is directly correlated with the development of Th1 (IFN- γ, IL-2 and TNF-α), or Th2 (IL-4, IL-5, IL-10, IL-13 and TGF-β) immune responses respectively, and the degree of immune activation is thought to directly impact the severity of disease^8^,^9^. Studies addressing the importance of other mediators, such as eicosanoids, Super Oxide Dismutase (SOD) and chemokines are few, especially in dogs^10^,^11^. Recently, our group has found the role played by LTB4 and PGE2 in modulating *Leishmania* infection in humans^11^,^12^. Experiments *in vitro* with human macrophages demonstrated that SOD increased the parasite burden in these cells due to the inhibition of reactive oxygen species (ROS)^10^.

Knowledge of their role in CVL will further our understanding of the complex pathogenicity of the disease. Moreover, dogs constitute a model to study VL, since clinical signs in this specie have some similarities to those developed in humans^13^,^14^, allowing its use in the study of new targets for prophylactic and therapeutic strategies.

It has been demonstrated that the production of anti-saliva antibodies in humans naturally exposed to *Lu. longipalpis* sand flies positively correlated with the development of delayed type hypersensitivity against *Leishmania*, and that in this setting, these antibodies are reported as a marker of protection against infection^15^. Notably, although dogs from endemic areas produce anti-saliva antibodies, there are no studies investigating whether these antibody levels correlate with protection, risk of disease development or transmission to sand flies.

Nonetheless, due to the complexity of CVL, there are difficulties in establishing a clear association between clinical manifestations and immunologic status. Herein, we hypothesize that only a single parameter of cellular or humoral immune responses cannot clearly define disease severity. Integrated studies of multiple biomarkers are needed to better understand their role in the outcome of *L. infantum* infection. In this cross sectional exploratory study, we identified a distinct biosignature in dogs with different clinical scores where an increase in the severity of disease was characterized by a continuous decrease in levels of LTB4 and PGE2 and an increase in levels of CXCL1 and CCL2. Additionally, using 3 different parameters (LTB4, PGE2 and CXCL1) we were able to discriminate between different clinical score ranges through the construction of ROC curves. Moreover, there is an augment in the frequency of dogs displaying anti-saliva IgG and high parasite load along with the increase of the clinical score. This study allows the evaluation of multiple biomarkers in dogs, which could be important for CVL surveillance in endemic areas.

## Results

### Expression of immune and inflammatory markers

After diagnosing CVL in the canine random sample 21.4% (15/70) were found to be negative for CVL whereas 78.6% (55/70) animals were infected. Clinical score evaluation on the infected dogs classified 40% (22/55) dogs with subclinical disease, 38.2% (21/55) with mild disease, and 21.8% (12/55) with severe disease.

**Figure 1:**
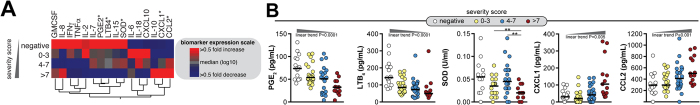
Distinct expression of immune and inflammatory markers in serum from dogs presenting with different VL clinical severity scores. (**A**) Hierarchical cluster analysis (Ward’s method) with bootstrap was performed to depict the overall expression profile of the indicated serum biomarkers in the different study groups. (**B**) Scatter plots of biomarkers displaying significant statistical differences (P < 0.005) between the study groups using Kruskal-Wallis test. Non-parametric linear trend *ad hoc* tests were employed to examine the variation of the biomarker levels following the clinical severity score. SOD levels did not exhibit linear trend, thus data were compared using the Dunn’s multiple comparisons test (**P < 0.01).

All the biomarkers were analysed independently using univariate statistical analyses corrected for multiple observation, and only those ones that displayed significant differences among the different clinical groups were considered for the further analysis. A hierarchical clustering analysis of immune and inflammatory profiles in serum from dogs with different CVL clinical scores underlined a distinct biosignature associated with increased disease severity (Fig. 1). Remarkably, animals with higher severity scores (4–7 and >7) exhibited heightened serum concentrations of IL-10, CXCL1 and CCL2, whereas those with lower clinical scores (0–3) displayed increased levels of IL-6, IL-18 and CXCL10 relative to the average values of the entire study population (Fig. 1A). Infected dogs displayed reduced levels of several other biomarkers of inflammation and oxidative stress (Fig. 1A) when compared to uninfected ones. Amongst all the biomarkers, PGE2 and LTB4 values displayed a linear trend that decreased with disease severity (Fig. 1B). Conversely, we observed an upward linear trend in the amounts of CXCL1 and CCL2 with increasing clinical scores (Fig. 1B). In addition, dogs with a clinical score range from 4 to 7 displayed the highest serum levels of SOD, while those with the highest severity scores (>7) exhibited the lowest concentrations of this enzyme (Fig. 1B).

### Network analysis of the circulating biomarkers in dogs

**Figure 2:**
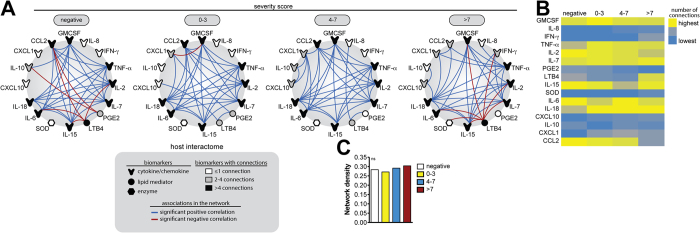
Network analysis of the circulating biomarkers in dogs with VL reveals a distinct biosignature of VL clinical severity. **(A)** The network analysis (host interactome) shows statistically significant Spearman correlations (P < 0.05) between of the biomarkers measured. See Supplemental File 1 for additional details on the strength (r value) and level of significance (P-value) for each individual correlation. **(B)** Heatmap of the number of statistically significant correlations involving each biomarker measured in dogs with different VL disease severity is shown. Details on the numbers of correlations are shown is Supplemental File 1. **(C)** Comparisons of the network densities is shown (density calculations are described in Methods). Data was compared using permutation test. ns, non significant.

We next examined the relationships between the biomarkers within each clinical group using network analysis based on statistically significant Spearman correlations (P < 0.05). We observed that the correlations profile exhibited distinct characteristics in each study group (Fig. 2A and Supplemental File 1). In addition, in all groups, most of the observed statistically significant correlations were positive (Fig. 2A and Supplemental File 1). Increased frequency of significant negative correlations was detected in the group of uninfected animals as well as in dogs with the highest severity scores (Fig. 2A). Of note, in these two groups of dogs, the majority of statistically significant correlations in the networks involving LTB4 were negative associations (Fig. 2A). Moreover, following quantification of the number of significant correlations observed in each marker (node analysis), we found that the overall number of connections involving most of the markers was similar between the clinical groups (Fig. 2B and Supplemental File1). Interestingly, animals with the highest severity scores (>7) displayed increased participation of LTB4 while showing a decrease in the involvement of CCL2 in the network (Fig. 2B). Additional analyses revealed that the network densities were not significantly different between the groups (Fig. 2C).

### ROC curve analyses using inflammatory markers to predict VL disease severity

**Figure 3:**
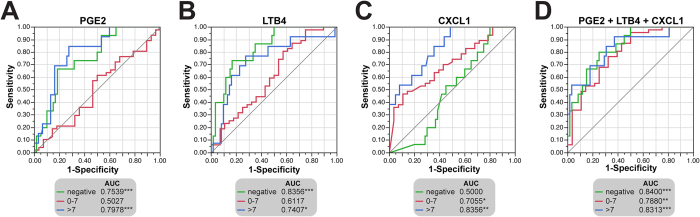
Using inflammatory markers to predict VL disease severity. **(A–D)** ROC curve analyses were performed to estimate in a quantitative way the performance of the different combinations of biomarkers used in the cluster analysis in segregating dogs diverging in VL clinical severity. AUC: area under the curve. *P < 0.05; **P < 0.01; ***P < 0.001.

ROC curve analyses were performed to estimate in a quantitative way the efficacy of different combinations of biomarkers in segregating dogs diverging in CVL clinical severity (Fig. 3). To simplify the analyses, the study groups were rearranged into: negative, score of 0–7 and score >7. We attempted to create a ROC curve combining the biomarkers that displayed a linear trend with disease severity score ranges (PGE2, LTB4, SOD, CXCL1 and CCL2, depicted in Fig. 1B). However, after testing different combinations (data not shown), the use of three biomarkers (PGE2, LTB4, and CXCL1) was shown to be the most efficient in distinguishing the clinical groups and therefore kept in the final ROC curve model. Figure 3A–C shows ROC curves for each marker PGE2, LTB4 and CXCL1, demonstrating that these three markers, when considered individually, have potential to distinguish the clinical groups. Notably, the combination of these biomarkers resulted in a better overall performance to discriminate the three clinical score categories (Fig. 3D).

### Associations between antibodies production against salivary recombinant proteins LJM11 and LJM17 and parasite load of infected dogs

**Figure 4:**
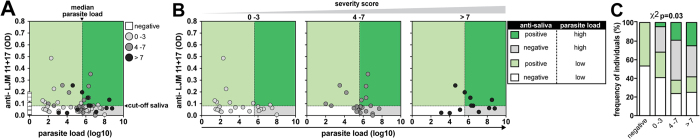
Associations between antibodies production against salivary recombinant proteins LJM11 and LJM17 and parasite load of infected dogs. (**A**) Correlations between parasite load and antibodies production against salivary recombinant proteins LJM11 and LJM17 in negative and infected dogs showing different clinical signs (n = 70). Dotted lines on the X-axis represent the median value of parasite load within the group of infected dogs, while dotted lines on Y-axis indicate the cut-off value for antibodies production against LJM11 and LJM17. In (**B**), the correlations were stratified according to clinical score presented by the dogs. White areas designate the quadrants that include the dogs displaying values of parasite load below the median and negative antibodies production; light green area designate the quadrants that include the dogs displaying values of parasite load below the median and positive antibodies production; dark green areas designate the quadrants that include the dogs displaying values of parasite load above the median and positive antibodies production; and grey areas designate the quadrants that include the dogs displaying values of parasite load above the median and negative antibodies production. In (**C**) the percentage of individuals within each area were compared between the groups with different clinical score using a chi-square analysis.

The associations between antibody production against recombinant sand fly salivary proteins LJM11 and LJM17 and parasite load of infected dogs is presented in Fig. 4A–C. There was no correlation between antibody concentrations against these recombinant salivary proteins and parasite loads in dogs (Fig. 4A). We observed an increase in the frequency of dogs showing high parasite load and anti-saliva antibodies with the increase of severity disease (Fig. 4B,C dark green bars). Although it seems to be an increase in the frequency of dogs showing negative saliva serology and high parasite loads, the results were not statistically significant among the groups (Fig. 4B,C grey bars).

## Discussion

In the present study, we evaluated different parameters associated to CVL severity, considering distinct groups of naturally infected dogs from a highly endemic area, in Camaçari, Bahia, Brazil. Our data revealed a distinct pattern of biomarkers correlated with the different clinical manifestations of CVL. We have also demonstrated that ROC curve analyses using combined biomarkers are able to distinguish dogs exhibiting different degrees of CVL severity. Furthermore, we identified that higher titers of antibodies to sand fly saliva is more frequent in highly parasitized dogs.

Cross sectional analysis of serum levels of cytokines and chemokines as well as LTB4 and PGE2 in dogs suggest that the relationships between the biomarkers in the study groups, and especially those related to LTB4 and CCL2, could be directly involved in the pathogenesis of CVL. Levels of LTB4 and PGE2 displayed a significant decrease following a gradual increase in disease severity. Recently, our group has shown the importance of eicosanoids in modulating immune responses in *Leishmania* infection^12^. In addition, PGE2 has been shown to contribute to parasite proliferation^11^,^16^, whereas LTB4 increases macrophage activation and intracellular destruction^17^,^18^,^19^. Therefore, the balance between these two mediators seems to contribute to the control of *Leishmania* infection and inflammation. The decrease in the serum levels of both LTB4 and PGE2 found in our work indicate that dogs displaying severe disease may loose the ability to mount an efficient response to control the infection.

SOD-1 has been found to play a deleterious role in *Leishmania* infection, increasing parasite burden in *Leishmania*-infected human macrophages^10^. In our samples, an increase in SOD was observed in the group of dogs with clinical score between 4 and 7, indicating that this enzyme could be related to the pathogenesis of disease. However, dogs with the highest clinical score displayed low levels of this enzyme, possibly reflecting exhaustion of the immune system.

In the context of experimental or natural CVL infection, an up-regulation of chemokines expression in the spleen has been already described, although only CXCL10 and CCL5 were shown to be markedly elevated in oligosymptomatic dogs^20^. Concerning these chemokines, no significant differences in study groups were noticed. However, our findings showed a significant increase in CXCL1 and CCL2 serum levels with the increase of disease severity. CXCL1 is responsible for the recruitment of neutrophils while CCL2 is responsible for the recruitment of monocytes^21^,^22^. Results from literature have shown that dogs with CVL display neutropenia^23^,^24^ and that derangement in neutrophil numbers and function seems to be important features of CVL^25^,^26^. Moreover, Menezes-Souza *et al*.^20^ reported a higher expression of CCL2 in the skin of dogs with CVL that positively associated with a higher parasite density. The authors also reported an increase in the number of macrophages in the skin of symptomatic dogs displaying high parasitism. Our results as well as others studies suggest that these chemokines are recruiting immature or unresponsive macrophages, since animals with higher serum level of CXCL1 and CCL2 presented high levels of parasite in the spleen.

We identified a signature of CVL characterized by the absence of inflammatory mediators such as IFN-γ, TNF-α, IL-2, IL-7, PGE2, LTB4, IL-15 and SOD in dogs with severe CVL. Although dogs with subclinical disease do not present many of the mediators described above, an observed increase in the levels of IL-6 and IL-18, suggests a restricted ability to control the infection. On the other hand, symptomatic dogs with a score higher than 7 showed a near absence of inflammatory mediators and an increase in IL-10, CCL2 and CXCL1 levels. Cytokines such as IFN- γ and TNF- α are considered the hallmark of protection in different clinical manifestations of leishmaniasis^8^. However, interactomes analysis showed that these protective cytokines do not have any correlation with the clinical status of the dogs, although in our study their levels decrease along the clinical score progression, but not in a significant way.

ROC curve analysis ultimately compared the overall performance of the diverse combination of candidate biomarkers in distinguishing different clinical manifestations of CVL. The combination of PGE2, LTB4 and CXCL1 was the one that better discriminated among the groups of animals, pointing out the importance of these mediators in the follow up and prognostic of clinical manifestations in CVL.

Salivary recombinant proteins are of value as markers of vector exposure. In humans and dogs, LJM11 and LJM17 emerged as potential markers of specific exposure to *Lu. longipalpis*^27^. Additionally, Gomes *et al*.^15^ positively correlates the appearance of an anti-saliva humoral response to an anti-*L. infantum* cell-mediated immunity raising the hypothesis that induction of immune response against sand fly saliva can facilitate the generation of a protective response against human VL. Thus, we can infer that anti-sand fly saliva antibodies can serve as an important epidemiological marker of vector exposure in endemic areas and even as a surrogate marker of protection. However, we observed an increase in the frequency of dogs with high parasite loads and anti-saliva antibodies with the increased of clinical scores. The strength of this dog study is the parasite load comparison together with the results of saliva serology. These type of data allowed the observation of the increased percentage of the highly infected and highly saliva exposed group along with disease severity. We speculate that dogs are likely more exposed to sand-fly bites in the endemic areas than humans, leading to an increase in the antibody production and higher chances of infection and elevated parasite load.

This study evaluated multiple biomarkers in dogs and defined a biosignature related to different clinical manifestations of CVL. Characterization of disease severity in CVL is essential to prevent the spread of the infection, since symptomatic animals are better disseminators of parasites in endemic areas. Further studies are in progress to define a particular biosignature for dogs that transmit parasites to sand flies allowing for better surveillance in areas of CVL transmission.

## Methods

### Study design

A random sample of 70 dogs was selected from a cross-sectional study conducted in the municipality of Camaçari, located in the State of Bahia, North-eastern Brazil (latitude: 12° 41′ 51′′S; longitude: 38° 19′ 27′′W). This area is endemic for both VL and CVL, with a seroprevalence of CVL ranging from 20 to 40% in the canine population (unpublished data).

### Ethical aspects

This study was approved by the IGM - FIOCRUZ Institutional Review Board for Animal Experimentation under Permit Number 007/2013, within Brazilian Federal Law on Animal Experimentation (Law no. 11794), and following the guidelines for animal research established by the Oswaldo Cruz Foundation. All the dog owners signed an informed consent form, allowing the examination of the animals. Dogs were grouped according to CVL diagnosis and clinical score, as explained below.

### CVL diagnosis

Serum and splenic aspirate samples were collected as described before^28^ and used to perform anti–sand fly saliva serology and biomarkers analyses.

Parasitological evaluation of splenic aspirates was performed as previously described^29^, as well as the use of qPCR technique to assess positivity in the splenic aspirate^28^. The splenic parasite load was measured by qPCR technique as described elsewhere^28^. Anti-*Leishmania* antibodies were detected using the double pathway platform-screening test (DPP^®^ CVL, Bio-Manguinhos Unit, Rio de Janeiro, Brazil) followed by the confirmatory immunoassay (EIE^®^ CVL, Bio-Manguinhos Unit, Rio de Janeiro, Brazil).

Dogs were considered infected if they present positive results in splenic culture or qPCR. Animals were considered uninfected if the above-mentioned tests were negative.

### Composite clinical severity score for CVL

All animals were clinically examined and classified according to the parameters described in Table 1. Each clinical sign was given a grade of 0, 1 or 2 depending on the intensity of the clinical manifestation. The clinical score was calculated as the sum of grades for each clinical sign with same weights. The composite score could then range from 0 to 24 points. Infected animals with a clinical score of ≤3 were classified as without clinical disease (subclinical); a clinical score of ≥4 < 7 were categorized as with mild disease and a clinical score of ≥7 was used to define severe disease.

### Immunoassays

Cytokine and chemokine levels in serum were measured using a pre-defined luminex-based multiparametric kit (Milliplex Map Kit - canine cytokine magnetic bead panel, Life Technologies, Carlsbad, CA, USA). The markers examined were IFN-γ, IL-10, TNF-α, IL-2, IL-6, IL-7, IL-15, IL-8, CCL2, CXCL10, GM-CSF and CXCL1. Concentrations of PGE2, LTB4 and superoxide dismutase (SOD) were measured in serum samples from all the dogs using an enzyme-linked immunoassay (Cayman Chemical, Ann Harbor, MI, USA) as described previously^10^,^12^.

### Anti–sand fly saliva serologic testing

Recombinant *Lu. longipalpis* salivary proteins were expressed in HEK cells and HPLC purified as previously described^27^. Anti–sand fly saliva serologic test ELISA was performed as described elsewhere^27^,^30^ with some adaptations for use in dogs. Antibody production against recombinant salivary proteins LJM11 and LJM17 was measured using the optical density values divided by the cut-off value of each experiment.

### Statistical Analysis

Median values with interquartile ranges (IQR) were used as measures of central tendency. The chi-square test was employed to compare frequencies between the study groups. Hierarchical cluster analysis (Ward’s method) with bootstrap was performed to depict the overall expression profile of serum biomarkers in the negative and the different severity score groups. Significant statistical differences between groups of varying severity scores were evaluated using the Kruskal-Wallis test. Non-parametric linear trend *ad hoc* tests were employed to examine the variation of all biomarker levels following the clinical severity score. Biomarkers that did not exhibit linear trend were compared using the Dunn’s multiple comparisons test.

Network analysis (host interactome) were generated from Spearman correlation matrices containing values of each biomarker measured in the serum samples, as described by Mendonça *et al*.^31^. The values were input in JMP 10.0 software (SAS, Cary, NC, USA). A heat map of the number of statistically significant correlations involving each biomarker was constructed for different severity score groups. Aiming to analyse the structure of the biomarker networks, the network density was calculated as described by Mendonça *et al*.^32^.

Receiver-Operator Characteristic (ROC) curve analyses were used to test the power of some biomarkers (PGE2, LTB4, SOD, CLCX1 and CCL2) and their combination to distinguish dogs presenting with different stages of CVL clinical severity. For this analysis, the severity score groups 0–3 and 4–7 were combined. ROC curve analyses were performed using JMP 10.0 software. A *p*-value below 0.05 was considered statistically significant.

Correlations between parasite load and antibodies production against salivary recombinant proteins LJM11 and LJM17 in negative and infected dogs showing different clinical signs were evaluated. The median values of parasite load and the cut-off value for antibodies production against LJM11 and LJM17 were used as measures of central tendency, differentiating high and low parameter levels. The *X*^2^ or Fisher tests were used to compare variables displayed as percentage.

## Additional Information

**How to cite this article**: Solcà, M. S. *et al*. Circulating Biomarkers of Immune Activation, Oxidative Stress and Inflammation Characterize Severe Canine Visceral Leishmaniasis. *Sci. Rep.* **6**, 32619; doi: 10.1038/srep32619 (2016).

## Supplementary Material

Supplementary Information

Supplementary File 1

## Figures and Tables

**Table 1:** VL clinical parameters employed to calculate the clinical score of each animal included in the study.

Clinical signs	Score based on intensity		
	0	1	2
Nutritional status	Normal or obese	Emaciate	Cachectic
Mucosa color	Normal	Anemic	—
Periocular dermatitis	Absent	Around one eye	Present in two eyes
Crust on ears	Absent	Present in one ear	Present in two ears
Ear Ulcers	Absent	Present in one ear	Present in two ears
Muzzle Depigmentation	Absent	In less than 1/3 of the muzzle	In more than 1/3 of the muzzle
Muzzle Hyperkeratosis	Absent	In less than 1/3 of the muzzle	In more than 1/3 of the muzzle
Muzzle Lesions	Absent	Initial mucous lesion	Larger ulcerated lesion
Spleen size	Not palpable	Enlarged	—
Onychogryphosis	Absent	Slight enlargement	Excessive enlargement
Alopecia	Absent	Focal	In more than 1/3 of the body
Seborrheic dermatitis	Absent	Focal	In more than 1/3 of the body
Lymphadenomegaly	Absent	One or two enlarged lymph nodes of the same pair	Enlarged lymph nodes of different pairs
