# Supplementary material for: Circulating Biomarkers of Immune Activation, Oxidative Stress and Inflammation Characterize Severe Canine Visceral Leishmaniasis
Source: Sci Rep. 2016 Sep 6;6:32619. doi: 10.1038/srep32619 (PMC5011641; doi:10.1038/srep32619)
Supplement: Supplementary Information [file srep32619-s1.doc]

**Supplementary Information**

**Manuscript title:**

Circulating Biomarkers of Immune Activation, Oxidative Stress and Inflammation Characterize Severe Canine Visceral Leishmaniasis

**Author list**

Manuela S. Solcà, Bruno B. Andrade, Melissa Moura Costa Abbehusen, Clarissa R. Teixeira, Ricardo Khouri, Jesus G. Valenzuela, Shaden Kamhawi, Patrícia Torres Bozza, Deborah Bittencourt Mothé Fraga, Valeria Matos Borges, Patrícia Sampaio Tavares Veras, Claudia Ida Brodskyn

**Legend supplementary dataset**

Spearman correlations and *p*-values of the host interactome, as well as the node analysis.
